# Assessing the risk of bluetongue to UK livestock: uncertainty and sensitivity analyses of a temperature-dependent model for the basic reproduction number

**DOI:** 10.1098/rsif.2007.1110

**Published:** 2007-07-17

**Authors:** Simon Gubbins, Simon Carpenter, Matthew Baylis, James L.N Wood, Philip S Mellor

**Affiliations:** 1 Institute for Animal Health, Pirbright LaboratoryAsh Road, Pirbright, Surrey GU24 0NF, UK; 2 Faculty of Veterinary Clinical Science, University of LiverpoolLeahurst, Neston, Wirral, Cheshire CH64 7TE, UK; 3 Cambridge Infectious Diseases Consortium, Department of Veterinary MedicineUniversity of Cambridge, Madingley Road, Cambridge CB3 0ES, UK

**Keywords:** bluetongue, epidemiology, basic reproduction number, model, sensitivity analysis, uncertainty analysis

## Abstract

Since 1998 bluetongue virus (BTV), which causes bluetongue, a non-contagious, insect-borne infectious disease of ruminants, has expanded northwards in Europe in an unprecedented series of incursions, suggesting that there is a risk to the large and valuable British livestock industry. The basic reproduction number, *R*_0_, provides a powerful tool with which to assess the level of risk posed by a disease. In this paper, we compute *R*_0_ for BTV in a population comprising two host species, cattle and sheep. Estimates for each parameter which influences *R*_0_ were obtained from the published literature, using those applicable to the UK situation wherever possible. Moreover, explicit temperature dependence was included for those parameters for which it had been quantified. Uncertainty and sensitivity analyses based on Latin hypercube sampling and partial rank correlation coefficients identified temperature, the probability of transmission from host to vector and the vector to host ratio as being most important in determining the magnitude of *R*_0_. The importance of temperature reflects the fact that it influences many processes involved in the transmission of BTV and, in particular, the biting rate, the extrinsic incubation period and the vector mortality rate.

## 1. Introduction

Bluetongue virus (BTV) is a double-stranded RNA virus (Reoviridae: *Orbivirus*) that causes bluetongue (BT), a non-contagious, insect-borne infectious disease of ruminants. All ruminant species are able to be infected, but improved breeds of sheep and some species of deer tend to develop particularly severe clinical signs of disease. Owing to this and its ability to spread rapidly and without apparent warning, BT has been cited as one of the most important of the Diseases Notifiable to the OIE. Between 1998 and 2005, the virus expanded northwards in Europe in an unprecedented series of incursions, causing the most severe outbreak of this disease ever recorded and resulting in the deaths of over 1.5 million sheep (Mellor & Wittmann 2002; Purse *et al.* 2005). In August and September 2006, the first outbreaks of BT were reported in northern Europe in Belgium, France, Germany and The Netherlands (Albers *et al.* 2007).

This dramatic expansion has been attributed to the northward movement of the major Old World vector of the disease, *Culicoides imicola*, probably under the influence of climate change, and to the involvement of more northerly *Culicoides* species whose distribution overlaps that of *C. imicola* and extends across the whole of central and much of northern Europe, including UK (Mellor & Leake 2000; Purse *et al.* 2005; Carpenter *et al.* 2006). The extension of BTV further north in Europe than ever before, and the involvement of vectors which also occur throughout UK, suggests that there is a risk to the large and valuable UK livestock industry (Wittmann & Baylis 2000; Purse *et al.* 2005). Indeed, it has been shown already that *Culicoides* present in some areas of UK are capable of replicating BTV to transmissible levels (Carpenter *et al.* 2006). To date, however, the level of risk according to other epidemiological variables and how this risk varies temporally and geographically across UK has yet to be assessed.

The basic reproduction number, *R*_0_, provides a powerful tool when assessing the risk of disease invasion (Anderson & May 1986, 1991; Heffernan *et al.* 2005). This quantity is defined as the average number of secondary cases arising from the introduction of a single infected individual to an otherwise susceptible population. A disease is able to invade a host population (i.e. increase in frequency) only if *R*_0_>1 and, consequently, *R*_0_ provides a means to assess the level of risk posed by a disease. Vector-borne diseases present a particular problem when computing *R*_0_, owing to the need to consider the biology of the host, the disease agent and the vector. Previous studies have derived *R*_0_ for a number of vector-borne diseases including malaria (MacDonald 1957), trypanosomiasis (Rogers 1988), African horse sickness (Lord *et al.* 1996), dengue (Newton & Reiter 1992; Luz *et al.* 2003) and West Nile virus (Wonham *et al.* 2004; Cruz-Pacheco *et al.* 2005), but, to date, the basic reproduction number for BTV has not been considered.

In this paper, we developed a model for the transmission of BTV in a mixed population of cattle and sheep and used this to compute *R*_0_. Estimates for each parameter which influences *R*_0_ were obtained from the published literature. Wherever possible estimates were derived to reflect the UK situation and, in particular, UK vector species. Moreover, explicit temperature dependence was included for those parameters for which it had been quantified. Uncertainty and sensitivity analyses were carried out to identify the most influential parameters in *R*_0_ and, hence, those for which the most robust estimates are required.

## 2. Transmission model for BTV

To capture the transmission dynamics of BTV, it is essential to consider the dynamics of infection in the ruminant host species (mainly cattle and sheep) and the *Culicoides* vector. It is important to include both cattle and sheep as hosts: cattle harbour the infection usually without displaying clinical disease and, hence, provide a covert reservoir of BTV for the vector, while sheep are often afflicted by severe clinical signs of disease.

The cattle and sheep populations are assumed to be constant (*H*_*i*_), except for disease-associated mortality, and are subdivided into the proportions susceptible (i.e. uninfected), infected and recovered, denoted by *x*^(*i*)^, *y*^(*i*)^ and *z*^(*i*)^, respectively, where the superscript *i* indicates cattle (*C*) or sheep (*S*). To allow for a more general distribution for the duration of viraemia, the infected host population, *y*^(*i*)^, is subdivided into a number of stages, with newly infected hosts entering the first stage and then passing through each successive stage. If the mean time spent in each stage is 1/*nr*, the total length of time spent in the *n* classes follows a gamma distribution, with mean 1/*r* and variance 1/*nr*^2^ (Lloyd 2001). For an appropriate choice of *n*, this approach includes the common epidemiological assumption of an exponentially distributed infectious period (*n*=1) and a fixed infectious period (*n*→∞).

The vector population (*N*) is subdivided into the number of adult female midges that are susceptible (i.e. uninfected), latent (i.e. infected, but not infectious) and infectious, denoted by *S*, *L* and *I*, respectively. To allow for a more general distribution for the extrinsic incubation (i.e. latent) period (EIP), the latent class is subdivided into a number of stages in a similar approach to that described above for the duration of host viraemia. Once infectious, midges remain so for life. Adult males and immature (larval and pupal) stages are not considered as they do not blood feed and, hence, do not transmit BTV.

The dynamics of BTV infection in the cattle and sheep populations are described by the following set of linked differential equations:

(2.1) $$\begin{array}{c} \frac{\text{d}x^{\left(i\right)}}{\text{d}t}=-\lambda_{i}x^{\left(i\right)},\\ \frac{\text{d}y_{1}^{\left(i\right)}}{\text{d}t}=\lambda_{i}x^{\left(i\right)}-(n_{i}r_{i}+d_{i})y_{1}^{\left(i\right)},\\ \frac{\text{d}y_{j}^{\left(i\right)}}{\text{d}t}=n_{i}r_{i}y_{j-1}^{\left(i\right)}-(n_{i}r_{i}+d_{i})y_{j}^{\left(i\right)},\;j=2,\ldots ,n_{i},\\ \frac{\text{d}z^{\left(i\right)}}{\text{d}t}=n_{i}r_{i}y_{n}^{\left(i\right)}, \end{array}$$

where *i* denotes host species and *j* the infection stage. The duration of viraemia in each host species follows a gamma distribution with mean 1/*r*_*i*_ and variance 1/*n*_*i*_*r*_*i*_^2^ (i.e. there are *n*_*i*_ stages each of mean duration 1/*n*_*i*_*r*_*i*_) and *d*_*i*_ is the rate at which infected animals die of severe clinical disease. Host reproduction and natural host mortality are ignored because the natural lifespan is much longer than the duration of an outbreak or the lifespan of the vector. The force of infection for each host species, *λ*_*i*_, is given by

(2.2) $$\lambda_{i}=ba_{i}\frac{N}{H_{i}}\frac{I}{N},$$

which is the product of the probability of transmission from an infected midge to a host (*b*), the biting rate on the species (*a*_*i*_), the ratio of vectors to hosts (*N*/*H*_*i*_) and the proportion of bites which are from infectious midges (*I*/*N*). The biting rate on species *i* can be decomposed such that *a*_*i*_=*aϕ*_*i*_, where *a* is the reciprocal of the time interval between blood meals and *ϕ*_*i*_ is the proportion of bites on the species. The proportion of bites on each host species (*ϕ*_*i*_) is given by

(2.3) $$\phi_{i}=\frac{\alpha_{i}H_{i}}{\sum_{j}\alpha_{j}H_{j}},$$

where *α*_*i*_ is a measure of vector preference for host species *i* (Sota & Mogi 1989; Kelly & Thompson 2000). In the case of two host species (i.e. cattle and sheep), equation (2.3) can be simplified to

(2.4) $$\phi_{\text{C}}=\phi =\frac{H_{\text{C}}}{H_{\text{C}}+\sigma H_{\text{S}}},$$

while that on sheep is *ϕ*_S_=1−*ϕ*. The parameter *σ* is a measure of vector preference for cattle compared to sheep: if *σ*<1, vectors feed preferentially on cattle, but if *σ*>1, they feed preferentially on sheep. Rather than work with host and vector populations sizes, it is often more convenient to use the vector to host ratio, *m*_*i*_=*N*/*H*_*i*_, in which case the force of infection, *λ*_*i*_, is *baϕ*_*i*_*m*_*i*_*I*/*N* (cf. equation (2.2)) and the proportion of bites on cattle is *ϕ*=*m*_S_/(*m*_S_+*σm*_C_) (cf. equation (2.4)).

The dynamics of BTV infection in the vector population are described by

(2.5) $$\begin{array}{c} \frac{\text{d}S}{\text{d}t}=\rho N-\lambda_{\text{V}}S-\mu S,\\ \frac{\text{d}L_{1}}{\text{d}t}=\lambda_{\text{V}}S-(k\nu +\mu )L_{1},\\ \frac{\text{d}L_{j}}{\text{d}t}=k\nu L_{j-1}-(k\nu +\mu )L_{j},\;j=2,\ldots ,k,\\ \frac{\text{d}I}{\text{d}t}=k\nu L_{k}-\mu I, \end{array}$$

where the subscript *j* denotes the stage of the EIP; *ρ* is the rate of recruitment to the adult female midge population; the EIP follows a gamma distribution with mean 1/*ν* and variance 1/*kν*^2^ (i.e. there are *k* stages each of mean duration 1/*kν*); and *μ* is the vector mortality rate. The force of infection for vectors, *λ*_V_, is given by

(2.6) $$\lambda_{\text{V}}=\beta a\left(\phi \sum_{j=1}^{n_{\text{C}}}y_{j}^{\left(\text{C}\right)}+(1-\phi )\sum_{j=1}^{n_{\text{S}}}y_{j}^{\left(\text{S}\right)}\right),$$

which is the product of the probability of transmission from an infected host to a midge (*β*) and the biting rate on infectious hosts of each species.

Point estimates or plausible ranges for each parameter were derived from the literature (table 1). Point estimates for parameters relating to the duration of host viraemia were obtained by fitting gamma distributions to published data. Ranges for the reciprocal of the time interval between blood meals (*a*), the vector mortality rate (*μ*) and the EIP (1/*ν*) reflect the dependence of these parameters on temperature and, furthermore, temperature-dependent functions have been derived for these parameters (Gerry & Mullens 2000; Mullens *et al.* 2004; table 1). Ranges for the remaining parameters reflect uncertainty in their values.

## 3. Basic reproduction number for bluetongue

In the mathematical theory of epidemics, the basic reproduction number, *R*_0_, is defined as the dominant eigenvalue, *r*(*K*), of the next-generation operator, *K* (Diekmann *et al.* 1990; Heffernan *et al.* 2005). For the transmission model described by equations (2.1)–(2.6), the basic reproduction number is given by

$$R_{0}=r(FV^{-1}),$$

where *F* is a matrix reflecting the rate at which new infections arise (i.e. transmission from vector to host or host to vector) and *V* is a matrix reflecting the rate at which individuals enter or leave infection classes (either due to completion of an infection stage or death). The methods used to compute *R*_0_ are presented in the electronic supplementary material, appendix A, though it is not possible to derive an expression in terms of the parameters for the general model.

For the special case in which the duration of viraemia in cattle and sheep and the EIP follow exponential distributions (i.e. *n*_C_=*n*_S_=*k*=1), the basic reproduction number can be derived explicitly (see electronic supplementary material, appendix A). In this case, it is given by

(3.1) $$R_{0}=\sqrt{\frac{b\beta a^{2}}{\mu }\left(\frac{\nu }{\mu +\nu }\right)\left(\frac{m_{\text{C}}\phi^{2}}{r_{\text{C}}+d_{\text{C}}}+\frac{m_{\text{S}}{\left(1-\phi \right)}^{2}}{r_{\text{S}}+d_{\text{S}}}\right)},$$

where the parameters are summarized in table 1 (cf. Lord *et al.* 1996). Heuristically, expression (3.1) can be understood as follows. For each host species, an infected individual remains infectious for 1/(*r*_*i*_+*d*_*i*_) days, during which time it is bitten by susceptible midges on average *am*_*i*_*ϕ*_*i*_ times per day, a proportion, *β*, of which results in an infected midge. Thus, on average a single infected host will result in *βam*_*i*_*ϕ*_*i*_/(*r*_*i*_+*d*_*i*_) infected midges. The probability of a vector surviving the EIP to become infectious is *ν*/(*μ*+*ν*). It survives for 1/*μ* days, during which time it will bite susceptible hosts at a rate *aϕ*_*i*_, a proportion of which, *b*, will result in an infected host. Thus, on average a single infected midge will result in *baϕ*_*i*_(*ν*/*μ*+*ν*)/*μ* infected hosts. The basic reproduction number (3.1) is the geometric mean of the number of infected midges per infected host and the number of infected hosts per infected midge.

## 4. Uncertainty and sensitivity analyses

For the uncertainty and sensitivity analyses, replicated Latin hypercube sampling (LHS) was used to explore the parameters influencing the basic reproduction number, *R*_0_ (Blower & Dowlatabadi 1994; Sanchez & Blower 1997; Luz *et al.* 2003). The LHS results were used to compute the median and maximum value for *R*_0_, and the proportion of samples yielding *R*_0_>1. The distributions for each parameter which resulted in values of *R*_0_<1 or *R*_0_>1 were plotted to identify parameters associated with *R*_0_>1. The sensitivity of *R*_0_ to changes in each parameter was assessed by calculating the partial rank correlation coefficients (PRCCs). Full details of these methods are presented in the electronic supplementary material, appendix A. The analyses were implemented for three types of farm depending on species composition: cattle only, sheep only and both cattle and sheep. This reflects the different types of farm found in UK and allows the impact of the different host species on the basic reproductive number to be assessed in greater detail.

## 5. Results

For all farm types, values of *R*_0_>1 were associated with higher values of the probability of transmission from host to vector (*β*), the vector to host ratios (*m*_C_ or *m*_S_) and temperature (*T*; see electronic supplementary material, appendix B for the full results). However, the effect of the vector to host ratios was weaker for farms with both cattle and sheep. For the remaining parameters, there were few differences between the distributions of the parameters yielding *R*_0_<1 or *R*_0_>1 for any of the farm types.

Cattle-only farms yielded the highest values for *R*_0_ (assessed in terms of the median and maximum values, and the proportion of samples yielding *R*_0_>1), followed by sheep-only farms, then mixed cattle and sheep farms (table 2). This effect of farm type is shown by the differences in empirical cumulative distribution functions (CDFs) for *R*_0_ (figure 1*a*,*c*,*e*) and the distribution of *R*_0_ at different sampled temperatures (figure 1*b*,*d*,*f*). Although the magnitude of *R*_0_ differed according to farm type, its dependence on temperature did not (figure 1*b*,*d*,*f*). Below 10°C, *R*_0_ was zero because the extrinsic incubation rate is zero (table 1). Above 10°C, *R*_0_ increased to a peak value in the range 20–25°C, after which it decreased.

PRCCs were calculated between each input parameter and the corresponding value of the basic reproduction number (figure 2). For all farm types, the strongest correlation was between temperature, which influences biting rate, EIP and vector mortality rate, and *R*_0_, with a PRCC>0.7. Similarly, the probability of transmission from host to vector (*β*) was significantly, positively correlated with *R*_0_ (PRCC=0.3) for all farm types. For farms which had only a single host species, there was a significant positive correlation between the vector to host ratio and *R*_0_ (PRCC=0.3). However, the strength of correlation diminished when two host species were present on a farm, only that for cattle remained significantly different from zero (*p*<0.05). For the remaining parameters, any correlations were weak and typically did not differ significantly from zero (*p*>0.05).

## 6. Discussion

The basic reproduction number, *R*_0_, provides a quantitative framework in which to address the question of level of risk posed by a disease to a population. It allows the identification, first, of what needs to be known in order to assess risk and, second, which of these factors are most important in determining the magnitude of risk. In the case of the transmission model for BTV developed in this paper, (2.1)–(2.6), 12 parameters were needed to compute *R*_0_. For three of these parameters (biting rate, EIP and vector mortality rate), it was possible to include explicit temperature dependence (table 1). Uncertainty and sensitivity analyses identified temperature as the most important factor in determining the magnitude of *R*_0_, though the probability of transmission from host to vector and the ratio of vectors to both cattle and sheep were also important (figures 1 and 2). The importance of temperature in determining *R*_0_ reflects the number of temperature-dependent processes involved in the transmission of BTV: the biting rate (Mullens & Holbrook 1991; Mullens *et al.* 2004), the EIP (Gerry & Mullens 2000; Wittmann *et al.* 2002; Mullens *et al.* 2004) and the vector mortality rate (Gerry & Mullens 2000; Wittmann *et al.* 2002). Moreover, these three temperature-dependent parameters were identified as being important if they were included directly in the uncertainty and sensitivity analyses instead of temperature (results not shown).

As with all uncertainty and sensitivity analyses, these conclusions are valid only over the parameter ranges considered. Wherever possible, these ranges (table 1) were derived to reflect the situation in UK, but in many cases they were drawn from data for a range of countries and vector species. Parameters relating to infection in the host are likely to be robust, but those relating to the vector are likely to vary with species and climate. For some parameters (time interval between blood meals, probability of transmission from host to vector and vector mortality rate), estimates were obtained for UK *Culicoides* species (Birley & Boorman 1982; Carpenter *et al.* 2006), but in most cases they were derived from outbreak areas, notably the USA (principal vector species, *Culicoides sonorensis*) and the Mediterranean basin (*C. imicola*; see references in table 1 for details). Although most effort should focus on estimating the five key parameters (biting rate, EIP, vector mortality rate, probability of transmission from host to vector and the ratio of vectors to cattle and sheep) and, in particular, their dependence on temperature and other environmental factors, it should be noted that all parameters require more robust estimates and, in particular, estimates that are directly applicable to UK.

The biting rate has often been cited as a critical parameter for the transmission of vector-borne diseases (McDonald 1957; Anderson & May 1991; Dye 1992; Lord *et al.* 1996), largely because it influences transmission from both host to vector and vector to host. The biting rate for *Culicoides* midges is related to the time interval between blood meals (table 1), and increases with temperature (Mullens & Holbrook 1991; Mullens *et al.* 2004). Furthermore, Gerry *et al.* (2001) showed that the host biting rate (defined as the product of the biting rate and the vector to host ratio) varied seasonally, most probably reflecting the influence of temperature (Gerry & Mullens 2000). In addition, a threshold value for the host biting rate was identified below which no seroconversions were observed in sentinel calves (Gerry *et al.* 2001), which reflects the dependence of *R*_0_ on this parameter (see, for example, equation (3.1)).

The EIP for BTV exhibits strong temperature dependence (Gerry & Mullens 2000; Wittmann *et al.* 2002; Mullens *et al.* 2004), varying from 26 days at 15°C to 4 days at 30°C (Wittmann *et al.* 2002). However, these values for the EIP are based on laboratory experiments in which *Culicoides* were maintained at a constant temperature; yet temperatures will clearly fluctuate in the field. As BTV is not able to replicate to transmissible levels within the vector at low temperatures (Wittmann *et al.* 2002), it will be important to extend the model so that the temperature dependence of the EIP is incorporated in a more realistic way.

Relatively small changes in the survivorship of a vector can, in principle, greatly affect the transmission of a disease agent, such as BTV, which requires a period after infection of a vector before transmission is possible. Seasonal variation in survivorship has been demonstrated for several *Culicoides* species (Birley & Boorman 1982; Braverman *et al.* 1985; Gerry & Mullens 2000) and, in particular, the vector mortality rate has been shown to increase with temperature (Gerry & Mullens 2000; Wittmann *et al.* 2002). The vector mortality rate will determine the probability that a vector will survive the EIP. Similarly, the biting rate is related to the time interval between blood meals, and the probability of a vector surviving this period will depend on survivorship. Consequently, the importance of the vector mortality rate arises from its impact on these processes. Moreover, the temperature dependence of all three of these parameters means that temperature has a strong influence on the magnitude of *R*_0_ (figures 1 and 2).

Estimates for the probability of transmission from host to vector varied from 0.1 to 15% (table 1). The lower estimates were for field-caught *C. sonorensis* midges in the USA (Nunamaker *et al.* 1997; Gerry *et al.* 2001), while the higher estimates were for *Culicoides obsoletus* group midges caught in UK and subsequently fed on infected blood in the laboratory (Carpenter *et al.* 2006). The difference in the probability of transmission may reflect species or population differences in oral susceptibility to BTV, or may result from the methods used to determine competence. Moreover, viral titres in the host vary during the course of an infection (Goldsmit *et al.* 1975; Veronesi *et al.* 2005), which may result in changes in the probability of transmission to the vector (Bonneau *et al.* 2002). In the model, however, host viral titre is assumed to be constant throughout viraemia, which may result in the overestimation of the basic reproduction number.

For farms with only a single host species (either cattle or sheep), the vector to host ratio was an important determinant of the magnitude of the basic reproduction number, with higher ratios associated with higher values of *R*_0_ (figure 2*a*,*b*; cf. equation (3.1)). For farms with both host species, the positive association remained, but was weaker (figure 2*c*). Moreover, *R*_0_ tended to be highest on cattle-only farms, lower on sheep-only farms and lowest on mixed farms (table 2 and figure 1). This represents a dilution effect (Lord *et al.* 1996) because sheep are assumed to be a poorer host compared with cattle in terms of the duration of viraemia. There are, however, aspects to the impact of the number of cattle and sheep on the magnitude of *R*_0_, which are not apparent from the uncertainty and sensitivity analyses. In particular, adding cattle to a sheep farm can result in an increase in *R*_0_ over a limited range for the number of cattle introduced, beyond which *R*_0_ decreases as the vector to host ratio decreases. This effect depends on vector preference and the relative duration of viraemia in the two host species (see, for example, equation (3.1); see also Lord *et al.* (1996)).

The transmission model, (2.1)–(2.6), considers only a single vector. However, there are likely to be multiple species involved, which may differ in their ability to transmit BTV. Both the *C. obsoletus* and *C. pulicaris* groups of midges are found in UK (Carpenter *et al.* 2006) and have been implicated in the transmission of BTV (Torina *et al.* 2004; Purse *et al.* 2006). Variation in oral susceptibility to BTV infection has been shown in *C. obsoletus* group midges caught in different regions of UK (Carpenter *et al.* 2006), which could represent different proportions of those species within the group possessing different levels of competence. Furthermore, Torina *et al.* (2004) presented tentative evidence based on light-trap catches that there may be differences between the *C. obsoletus* and *C. pulicaris* groups in terms of vector abundance required for transmission to occur. These features could be incorporated in the model by extending it to include multiple vector species or, alternatively, to use location-specific parameter values based on local species composition.

There are 24 known serotypes of BTV (Mertens 1999) and, in this study, data for several serotypes have been combined when estimating certain parameters, for example, the duration of host viraemia or the EIP (see references in table 1 for details). However, there may be differences among serotypes and, in particular, interactions between host, virus and vector, which influence the ability of BTV to invade a host population. For example, BTV serotypes 1, 2, 4, 9 and 16 have all been introduced to Europe, but have not extended much beyond the northern rim of the Mediterranean basin (Mellor & Wittman 2002; Purse *et al.* 2005), while BTV serotype 8 has appeared and spread in northern Europe (Albers *et al.* 2007).

The principal motivation for computing the basic reproduction number, *R*_0_, was to provide a quantitative framework in which to assess the risk of BTV to UK livestock. Uncertainty analysis (figure 1) provides evidence that there is a risk (i.e. there were parameter combinations which yielded *R*_0_>1), which is perhaps unsurprising in the light of the recent BT outbreak in northern Europe (Albers *et al.* 2007). Furthermore, the analysis suggested that the risk is greatest when the temperature is between 15 and 25°C (figure 1*b*,*d*,*f*). At lower temperatures, BTV is unable to replicate to transmissible levels, while at higher temperatures the vector is less likely to survive for long enough to complete the EIP. However, the transmission model ignores a large number of heterogeneities which will influence both spatial and temporal variation in *R*_0_, for example, the distribution of ruminant and vector species, and regional differences in environmental factors, in particular, temperature. A full risk assessment will need to take these factors into account and produce spatio-temporal maps for *R*_0_.

In addition to its use in predicting risk, the basic reproduction number can also be used to inform control strategies. In particular, those measures which affect parameters identified as most important by the sensitivity analysis are most likely to have the greatest effect. Any measures which reduce the biting rate, for example, could potentially reduce transmission and, hence, *R*_0_. This could possibly be achieved through the use of dips or pour-ons (Mullens *et al.* 2000, 2001; Doherty *et al.* 2004) and, consequently, it is essential to estimate the reduction in biting rate that can be achieved when using these treatments to assess whether or not they are likely to be effective control measures during a BTV outbreak.

The focus of the current study has been the risk of an invasion of BTV occurring in British livestock and, as such, it does not address the question of whether or not BTV could become endemic in UK. The transmission model, (2.1)–(2.6), could be used to assess the risk of persistence, but to do so will require a greater understanding of the dynamics of the vector population. Moreover, consideration would need to be given to mechanisms by which BTV could overwinter, such as covert persistence in immune cells in the vertebrate host (Takamatsu *et al.* 2003) or persistence in the larval stages of the vector, following the transovarial transmission (White *et al.* 2005).

## Figures and Tables

**Figure 1:**
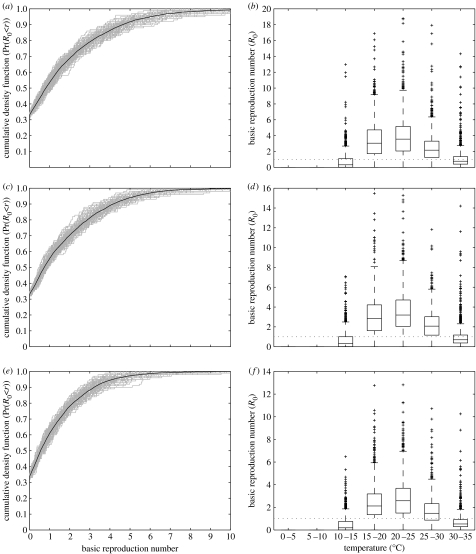
Uncertainty analysis of the basic reproduction number for farms with (*a*,*b*) cattle only, (*c*,*d*) sheep only, or (*e*,*f*) both cattle and sheep. (*a*,*c*,*e*) Empirical CDFs for the basic reproduction number. The grey lines are the CDFs for individual replicates, while the black line is the CDF for all replicates combined. (*b*,*d*,*f*) Box and whisker plots showing *R*_0_ and its dependence on temperature. The boxes show the lower quartile, median and upper quartile for *R*_0_; the whiskers indicate 1.5 times the interquartile range; and the crosses indicate any outlying values. Results in each figure are based on 100 replicates of the LHS scheme.

**Figure 2:**
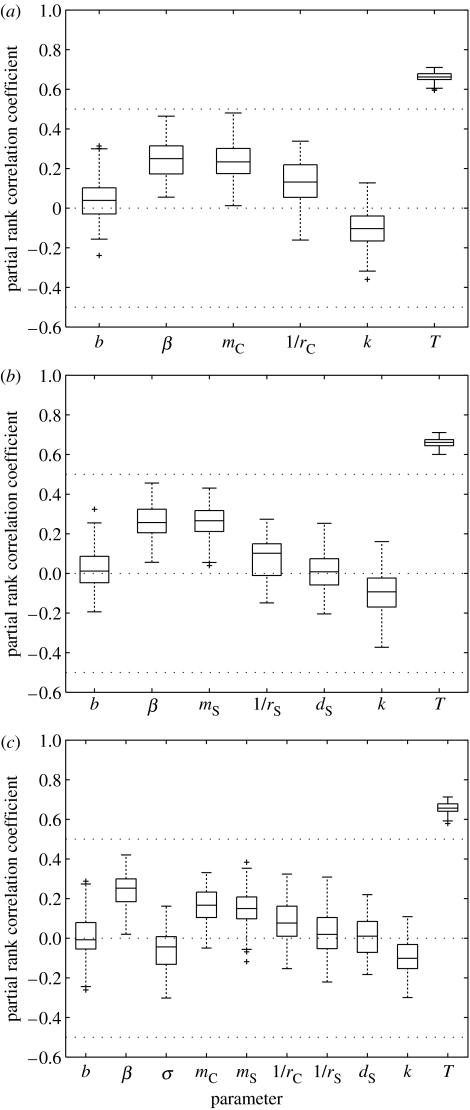
Box and whisker plots of the PRCC between each parameter (see table 1 for definitions) and the basic reproduction number for farms with (*a*) cattle only, (*b*) sheep only, or (*c*) both cattle and sheep. The boxes show the lower quartile, median and upper quartile for the PRCC; the whiskers indicate 1.5 times the interquartile range; and the crosses indicate any outlying values. Results are based on 100 replicates of the LHS scheme.

**Table 1:** Parameters in the transmission model for BTV.

description	symbol	estimate or range	comments	references
probability of transmission from vector to host	*b*	0.8–1.0	—	O'Connell (2002)
probability of transmission from host to vector	*β*	0.001–0.15	—	Nunamaker *et al.* (1997), Gerry *et al.* (2001) and Carpenter *et al.* (2006)
biting rate on species *i*	*a*_*i*_	—	can be decomposed so that *a*_*i*_=*aϕ*_*i*_ (see §2)	—
reciprocal of the time interval between blood meals	*a*	0–0.5	depends on temperature: *a*(*T*)=0.0002*T*(*T*−3.7)(41.9−*T*)^1/2.7^, Mullens *et al.* (2004)	Birley & Boorman (1982), Braverman *et al.* (1985), Mullens & Holbrook (1991) and Mullens *et al.* (2004)
proportion of bites on cattle	*ϕ*	—	see §2	—
vector preference for cattle compared to sheep	*σ*	0–1	vectors feed preferentially on cattle based on data for *C. imicola*	Nevill (1979) and Braverman *et al.* (2003)
ratio of vectors to cattle	*m*_C_	0–5000	based on a maximum host biting rate (*m*_*i*_*a*_*i*_) of 2500 bites per host per day; cf. median holding size of 60 breeding cattle (census data) and light-trap catches of 0–5000 midges per trap day (P. S. Mellor 1993, unpublished data, from Pirbright, UK)	Gerry *et al.* (2001)
ratio of vectors to sheep	*m*_S_	0–5000	see comments for vector to cattle ratio; cf. median holding size of 270 breeding ewes (census data) and light-trap catches of 0–5000 midges per trap day (P. S. Mellor 1993, unpublished data, from Pirbright, UK)	—
duration of viraemia (cattle)				
mean	1/*r*_C_	20.6	duration of viraemia based on natural infection and virus isolation in embryonated chicken eggs; parameters estimated by fitting a gamma distribution to data presented in paper referred to in right-hand column	Melville *et al.* (1996)
no. of stages	*n*_C_	5		
disease-induced mortality rate (cattle)	*d*_C_	0	cattle assumed not to succumb to severe disease; even for a naive population, as was the case for the BT outbreak in northern Europe in 2006, mortality in cattle was very low (0.1%), Albers *et al.* (2007)	Taylor (1986) and Melville *et al.* (1996)
duration of viraemia (sheep)				
mean	1/*r*_S_	16.4	duration of viraemia based on experimental infection and virus isolation in embryonated chicken eggs; parameters estimated by fitting a gamma distribution to data presented in papers referred to in right-hand column	Goldsmit *et al.* (1975) and Veronesi *et al.* (2005)
no. of stages	*n*_S_	14		
disease-induced mortality rate (sheep)	*d*_S_	0.001–0.01	derived from observed mortality in sheep ranging from 3.9 to 14.4%	Savini *et al.* (2005) and Albers *et al*. (2007)
vector recruitment rate	*ρ*	—	*R*_0_ does not include this parameter (see electronic supplementary material, appendix A)	
EIP				
mean	1/*ν*	4–26	depends on temperature: *ν*(*T*)=0.0003*T*(*T*−10.4), Mullens *et al.* (2004)	Gerry & Mullens (2000), Wittmann *et al.* (2002) and Mullens *et al.* (2004)
no. stages	*k*	1–100		
vector mortality rate	*μ*	0.1–0.5	depends on temperature: *μ*(*T*)=0.009 exp(0.16*T*), Gerry & Mullens (2000)	Birley & Boorman (1982), Braverman *et al.* (1985), Gerry & Mullens (2000) and Wittmann *et al.* (2002)

**Table 2:** Summary statistics for the uncertainty analysis.

farm type	*R*_0_		Pr(*R*_0_>1)

	median	maximum	
cattle only	0.81	18.77	0.47
sheep only	0.73	15.48	0.45
both cattle and sheep	0.55	12.82	0.39

## Supplementary Material

Electronic appendices Methods and full results
