# Supplementary material for: Assessing the risk of bluetongue to UK livestock: uncertainty and sensitivity analyses of a temperature-dependent model for the basic reproduction number
Source: J R Soc Interface. 2007 Jul 17;5(20):363–71. doi: 10.1098/rsif.2007.1110 (PMC2497440; doi:10.1098/rsif.2007.1110)
Supplement: Supplementary file 1 [file rsif20071110s03.pdf]

Assessing the risk of bluetongue to British livestock: uncertainty and sensitivity analysis of a temperature-dependent model for the basic reproduction number

Simon Gubbins[1,*], Simon Carpenter[1], Matthew Baylis[2], James L.N. Wood[3] and Philip S. Mellor[1]

[1] Institute for Animal Health, Pirbright Laboratory, Ash Road, Pirbright, Surrey GU24 0NF, U.K.

[2] Faculty of Veterinary Clinical Science, University of Liverpool, Leahurst, Neston, Wirral, Cheshire CH64 7TE, U.K.

[3] Cambridge Infectious Diseases Consortium, Department of Veterinary Medicine, University of Cambridge, Madingley Road, Cambridge CB3 0ES, U.K.

Electronic Appendix A Methods

This electronic appendix presents in detail the mathematical methods used to compute and analyse the basic reproduction number, R_0, for bluetongue virus (BTV).

1 Methods for computing R_0

In the mathematical theory of epidemics, R_0 is defined as the dominant eigenvalue, $r(K)$, of the next-generation operator, K (Diekmann *et al.* 1990; Heffernan *et al.* 2005). For the BTV transmission model described by equations (1)-(6), the next-generation operator is a matrix, K, which is given by,

$$K = FV^{-1},$$

and, hence, the basic reproduction number is given by,

$$R_0 = r(FV^{-1}),$$

where F is a matrix reflecting the rate at which new infections arise and V is a matrix reflecting the rate at which individuals enter or leave infection classes.

The matrix, F, is an n_C+n_S+k+1 by n_C+n_S+k+1 square matrix, whose elements, F_{ij}, give the rate at which new infections of type i arise from an infected individual of type j. (The types of infected individuals are ordered as cattle in infection stages 1 to n_C, sheep in infection stages 1 to n_S, vectors in latent stages 1 to k and infectious vectors, respectively.) Transmission only occurs between infectious vectors and susceptible hosts and between infected hosts and susceptible vectors. Consequently, all elements of F are zero, except for,

$$F_{1,n_C+n_S+k+1} = bam_C\phi,$$
$$F_{n_C+1,n_C+n_S+k+1} = bam_S(1-\phi),$$

which correspond to transmission from vectors to susceptible hosts, and

$$F_{n_C+n_S+1,j} = \beta a\phi, \qquad j = 1,\ldots,n_C,$$
$$F_{n_C+n_S+1,n_C+j} = \beta a(1-\phi), \qquad j = 1,\ldots,n_S,$$

which correspond to transmission from cattle and sheep in each infected stage, respectively, to susceptible vectors.

The matrix, V, is also an n_C+n_S+k+1 by n_C+n_S+k+1 square matrix, which has the following block structure,

$$V = \begin{pmatrix} A & 0 & 0 \\ 0 & B & 0 \\ 0 & 0 & C \end{pmatrix},$$

where A, B and C are n_C by n_C, n_S by n_S, k+1 by k+1 square matrices relating to infected cattle, sheep and vectors, respectively. The elements of A and B are given by,

$$A_{jj} = n_C r_C + d_C \qquad j = 1,\ldots,n_C,$$
$$A_{j+1,j} = -n_C r_C \qquad j = 1,\ldots,n_C-1,$$
$$B_{jj} = n_S r_S + d_S \qquad j = 1,\ldots,n_S,$$
$$B_{j+1,j} = -n_S r_S \qquad j = 1,\ldots,n_S-1,$$

with the remaining elements equal to zero. Each non-zero element corresponds to the rate at which infected hosts leave infection stage j (A_{jj} and B_{jj}) or move into infection stage j+1 from infection stage j ($A_{j+1,j}$ and $B_{j+1,j}$). The elements of C are given by,

$$\begin{aligned} C_{jj} &= k\nu + \mu \quad j = 1,\ldots,k, \\ C_{j+1,j} &= -k\nu \quad j = 1,\ldots,k, \\ C_{k+1,k+1} &= \mu, \end{aligned}$$

with the remaining elements equal to zero. Each non-zero element corresponds to the rate at which latent vectors leave incubation stage j (C_{jj}) due to death or completion of the stage, move into latent stage j+1 from latent stage j ($C_{j,j+1}$) or the rate at which infectious vectors die ($C_{k+1,k+1}$).

For the special case in which the duration of viraemia in cattle and sheep and the extrinsic incubation period follow exponential distributions (i.e. n_C=n_S=k=1), the matrices, F and V, are given by,

$$F = \begin{pmatrix} 0 & 0 & 0 & bam_C\phi \\ 0 & 0 & 0 & bam_S(1-\phi) \\ \beta a\phi & \beta a(1-\phi) & 0 & 0 \\ 0 & 0 & 0 & 0 \end{pmatrix},$$

and,

$$V = \begin{pmatrix} r_C + d_C & 0 & 0 & 0 \\ 0 & r_S + d_S & 0 & 0 \\ 0 & 0 & \nu + \mu & 0 \\ 0 & 0 & -\nu & \mu \end{pmatrix}.$$

Hence, the next generation matrix, K, is,

$$K = FV^{-1} = \begin{pmatrix} 0 & 0 & \dfrac{bam_C\phi}{\mu}\left(\dfrac{\nu}{\mu+\nu}\right) & \dfrac{bam_C\phi}{\mu} \\ 0 & 0 & \dfrac{bam_S(1-\phi)}{\mu}\left(\dfrac{\nu}{\mu+\nu}\right) & \dfrac{bam_S(1-\phi)}{\mu} \\ \dfrac{\beta a\phi}{r_C + d_C} & \dfrac{\beta a(1-\phi)}{r_S + d_S} & 0 & 0 \\ 0 & 0 & 0 & 0 \end{pmatrix}.$$

Some linear algebra shows that the dominant eigenvalue of K (i.e. R_0) is,

$$R_0 = \sqrt{\frac{b\beta a^2}{\mu}\left(\frac{\nu}{\mu+\nu}\right)\left(\frac{m_C\phi^2}{r_C + d_C} + \frac{m_S(1-\phi)^2}{r_S + d_S}\right)}.$$

2 Uncertainty and sensitivity analyses

For the uncertainty and sensitivity analyses, Latin hypercube sampling (LHS) was used to explore the parameters influencing the basic reproduction number, R_0 (Blower & Dowlatabadi 1994; Sanchez & Blower 1997; Luz *et al.* 2003). Probability density functions (PDFs) were defined for each parameter: Gamma distributions for the duration of viraemia in cattle and sheep; a uniform distribution for temperature ranging from 0°C to 35°C, with the temperature-dependent parameters (a,ν,μ; see table 1) calculated at each sampled

temperature; and uniform distributions for the remaining parameters with maximum and minimum values given in table 1. Each PDF was subdivided into 100 equiprobable intervals and then randomly sampled 100 times without replacement. The 100 samples from each PDF were then randomly permuted to yield 100 distinct parameter sets. For each parameter set, the corresponding value of R_0 was computed using the methods described above. Replicated LHS was then implemented by repeating this procedure 100 times.

The LHS results were used to compute the median and maximum value for R_0, and the proportion of samples yielding $R_0>1$. The distributions for each parameter which resulted in values of R_0 above or below one were plotted to identify parameters associated with $R_0>1$. The sensitivity of R_0 to changes in each parameter was assessed by calculating the partial rank correlation coefficients (PRCCs) using the method described in Blower & Dowlatabadi (1994). Approximate *t*-tests were used to determine whether or not the PRCCs differed significantly from zero (Blower & Dowlatabadi 1994). To assess the variability in the PRCCs, they were computed for each replicate of the Latin hypercube sampling.

References

Blower, S.M. & Dowlatabadi, H. 1994 Sensitivity and uncertainty analysis of complex models of disease transmission: an HIV model as an example. *Int. Stat. Rev.* **62**, 229-243.

Diekmann, O., Heesterbeek, J.A.P. & Metz, J.A.J. 1990 On the definition and the computation of the basic reproduction ratio R_0 in models for infectious diseases in heterogeneous population. *J. Math. Biol.* **28**, 365-382.

Heffernan, J.M., Smith, R.J. & Wahl, L.M. 2005 Perspectives on the basic reproductive ratio. *J. R. Soc. Interface* **2**, 281-293.

Luz, P.M., Codeco, C.T., Massad, E. & Struichner, C.J. 2003 Uncertainties regarding dengue modelling in Rio de Janeiro, Brazil. *Mem. Inst. Oswaldo Cruz* **98**, 871-878. (Available online from: http://www.scielo.br/scielo.php?script=sci_arttext&pid=S0074-02762003000700002&lng=en&nrm=iso).

Sanchez, M.A. & Blower, S.M. 1997 Uncertainty and sensitivity analysis of the basic reproductive rate: tuberculosis as an example. *Amer. J. Epidemiol.* **145**, 1127-1137.

Electronic Appendix B Uncertainty analysis

This electronic appendix presents the full results of the uncertainty analysis of the basic reproduction number, R_0, for bluetongue virus (BTV). The distributions for each parameter which resulted in values of R_0 above or below one were plotted to identify parameter values associated with R_0>1. The analyses were implemented for three types of farm depending on species composition: cattle only (figure S1); sheep only (figure S2); and both cattle and sheep (figure S3).

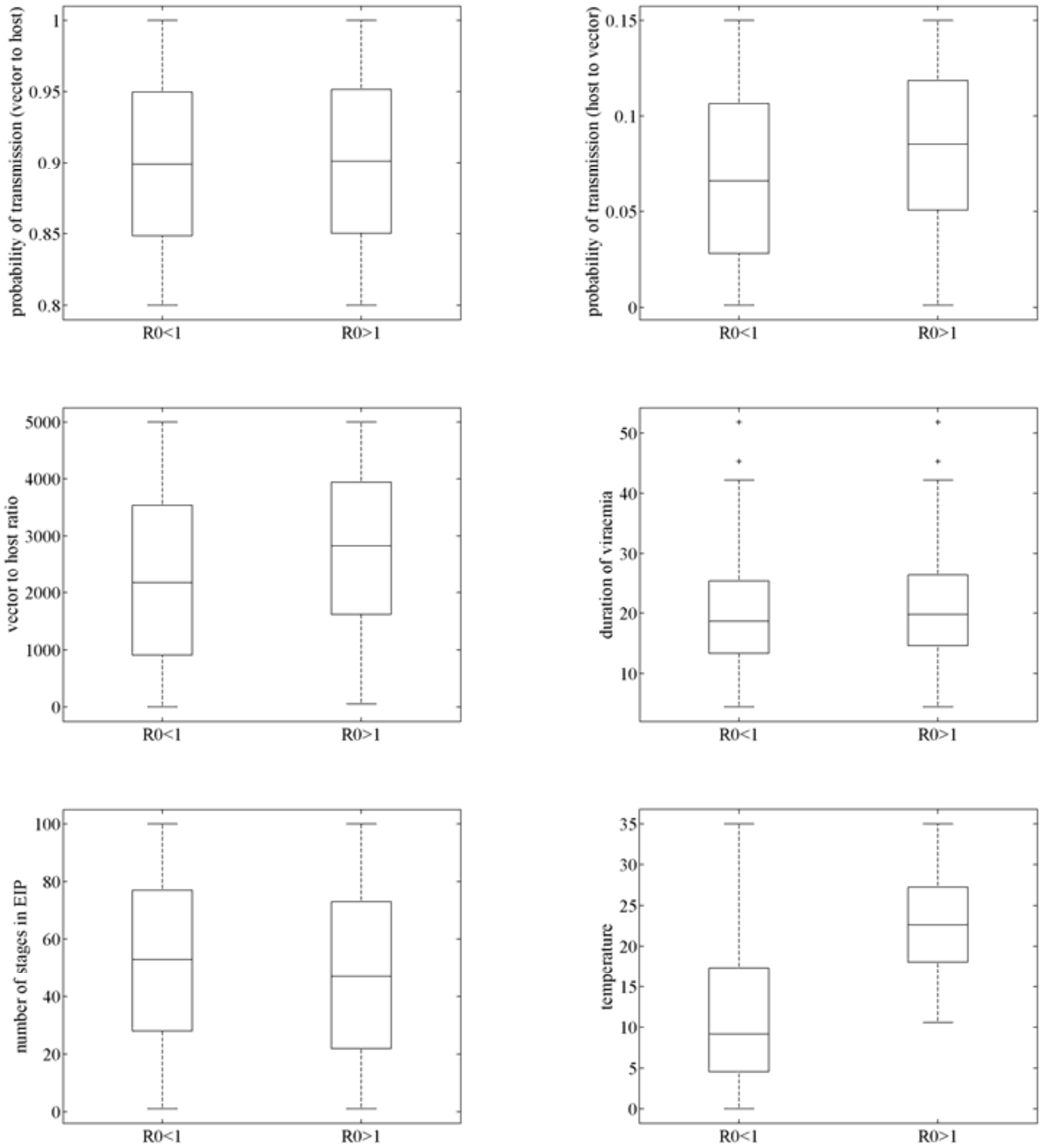

Figure S1. Uncertainty analysis of the basic reproduction number for BTV for farms with cattle only. Box and whisker plots showing the distribution of sampled parameter sets leading to disease extinction (R_0<1) or disease outbreaks (R_0>1). The boxes mark the lower quartile, median and upper quartile for the sampled parameters; the whiskers indicate 1.5 times the interquartile range; and the crosses indicate outlying values. Results are based on 100 replicates of the Latin hypercube sampling scheme.

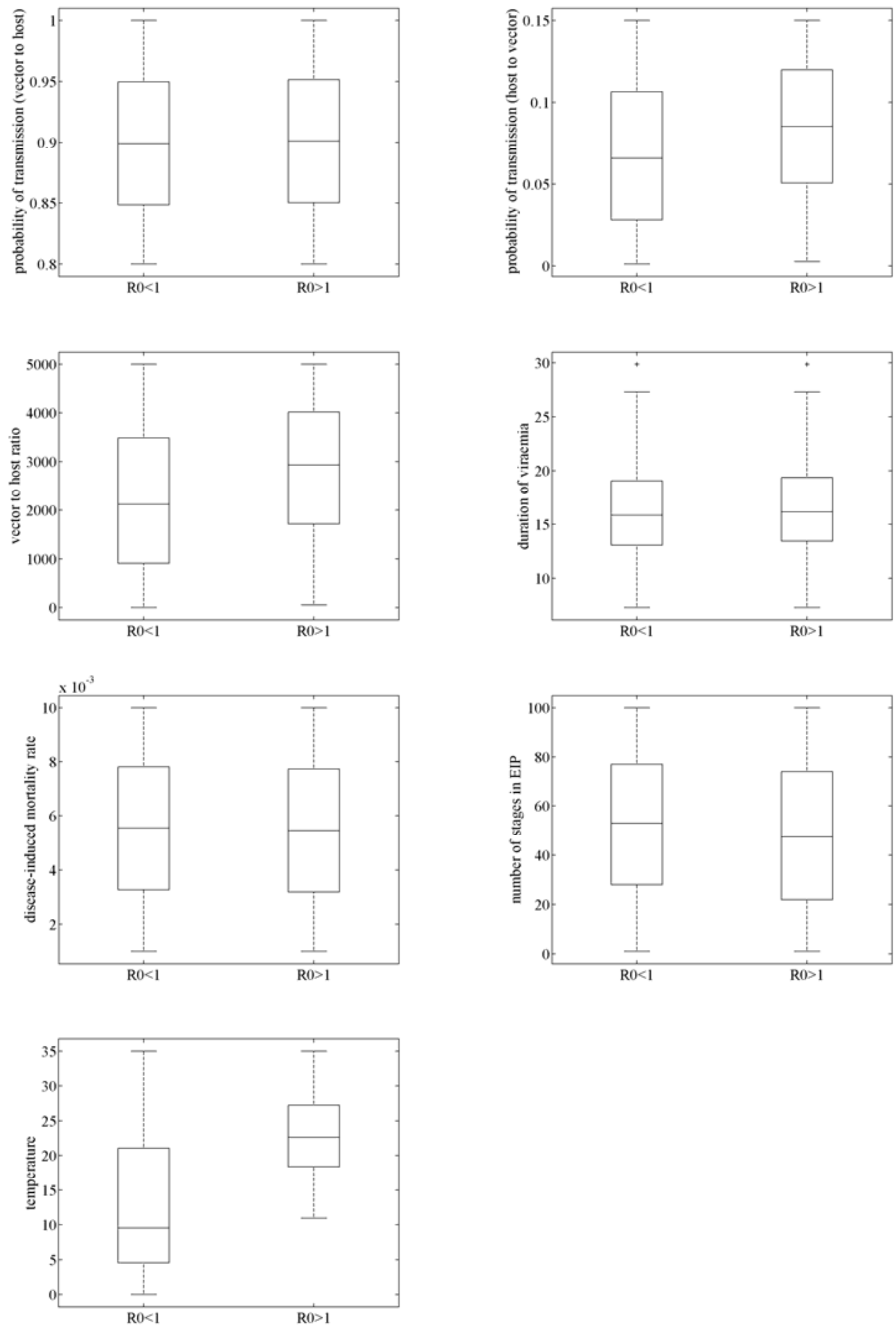

Figure S2. Uncertainty analysis of the basic reproduction number for BTV for farms with sheep only. Box and whisker plots showing the distribution of sampled parameter sets leading to disease extinction (R_0<1) or disease outbreaks (R_0>1). The boxes mark the lower quartile, median and upper quartile for the sampled parameters; the whiskers indicate 1.5 times the interquartile range; and the crosses indicate outlying values. Results are based on 100 replicates of the Latin hypercube sampling scheme.

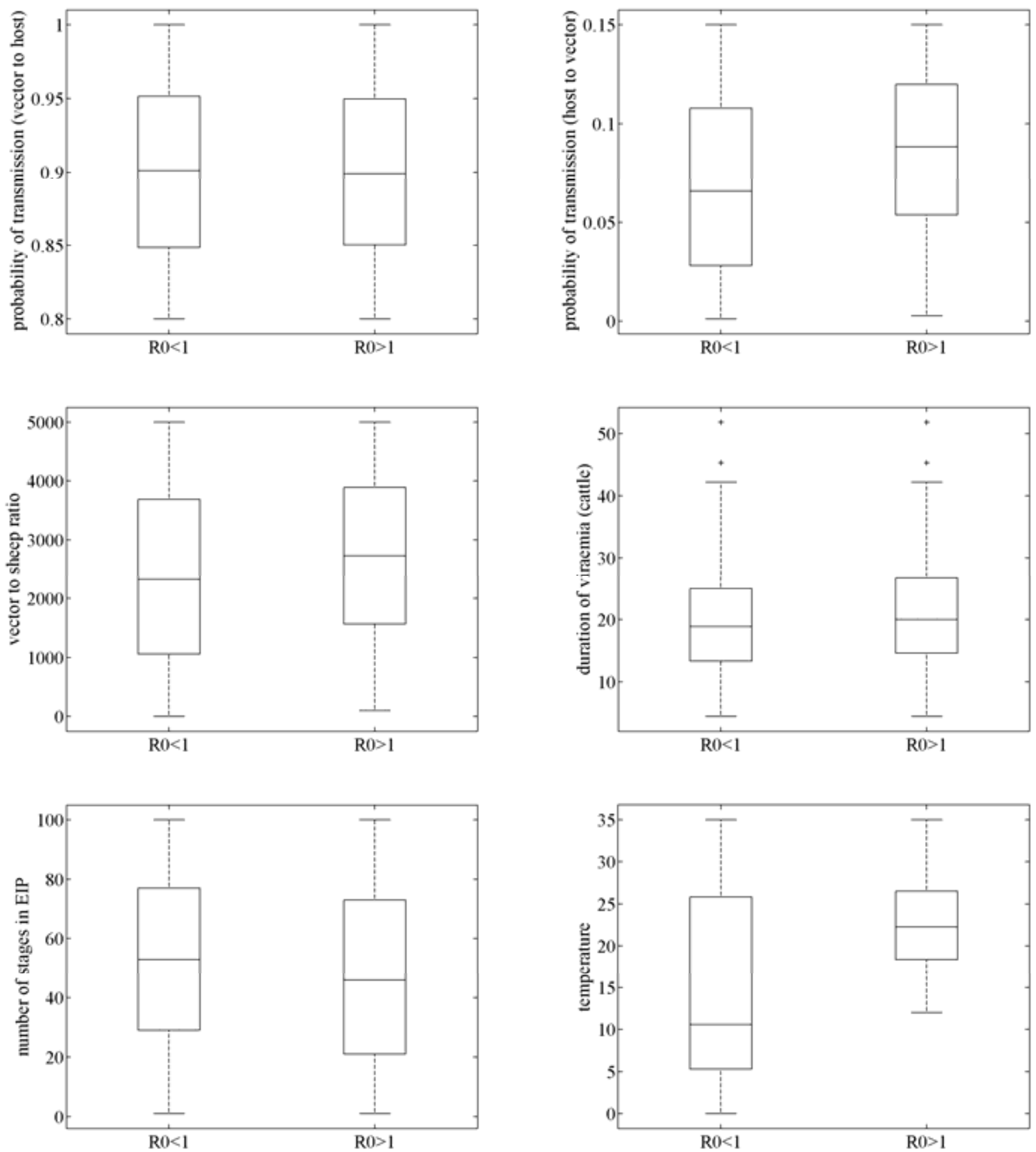

Figure S3. Uncertainty analysis of the basic reproduction number for BTV for farms with both cattle and sheep. Box and whisker plots showing the distribution of sampled parameter sets leading to disease extinction (R_0<1) or disease outbreaks (R_0>1). The boxes mark the lower quartile, median and upper quartile for the sampled parameters; the whiskers indicate 1.5 times the interquartile range; and the crosses indicate outlying values. Results are based on 100 replicates of the Latin hypercube sampling scheme.
